# The British Pharmacopœia

**Published:** 1885-12

**Authors:** 


### Notes on Books.

---

*The British Pharmacopœia.* London: SPOTTISWOODE and Co. 1885.

The General Medical Council resembles nothing so much as a female elephant confined in a menagerie. Its size is great, and its separate members individually imposing; its maintenance is highly expensive, and its rapacity large; its feeble acts ludicrously disproportionate to its capability, and its fecundity extraordinarily low! At length, after some years of gestation and a lingering labour, the Medical Council has been delivered of an offspring, of which, to tell the truth, if it has no reason to be ashamed, neither has it cause to be unduly proud.

The work will have been studied more or less closely by all practitioners ere this, and a detailed notice of it is, therefore, unnecessary; but we may be permitted to make a few remarks on some of the more striking features. Judged as a whole, the new edition is a marked advance upon its predecessor in respect of the more consistent and more scientific chemical nomenclature now employed, of improved pharmaceutical processes and preparations, of some omissions, and, most of all, in respect of the many additions.

The change in chemical nomenclature does not call for special remark from the medical point of view, but to the student it is a convenience to find substances called by the same names by his lecturers on Chemistry and Materia Medica respectively. The improvements in pharmaceutical processes and preparations are numerous, especially in preparations, and consist principally in the "standardising" of such galenical preparations as the extract and tincture of nux vomica, the liquid extract of cinchona, and cherry-laurel water: these now contain a definite percentage of their active ingredients, which will lead to their employment with increased certainty and satisfaction. Most of the liquors have been altered from the strength of 4 grains to the ounce to a solution of the strength of 1 per cent.: this is of no advantage whatever, and increasing the strength of solutions may lead to the occurrence of accidents; the solution of morphia for hypodermic purposes is in the same way increased in strength from 1 grain in 12 minims to 1 grain in 10 minims, and here the danger from overdose is still more likely. At all times a solution arranged upon a duodecimal base is more conveniently divisible than one upon a decimal base: had mankind, like certain giants, been furnished with six instead of five fingers, our numerical system would have been more generally convenient.

The formulæ for pulv. glycyrrhizæ co. and pulv. elaterini co. are improvements on those they replace: many other alterations, however, such as those of the preparations of phosphorus, are not less unsatisfactory than those in the late edition.

Of the substances omitted from the new Pharmacopœia, few will regret the absence of castoreum, ulmi cortex, dulcamara, and rhamni succus; but it is a serious error no longer to recognise as officinal the green iodide of mercury, so largely used in many different countries, and frequently tolerated when the red iodide cannot be borne. The leaves of stramonium also were valuable for smoking purposes in spasmodic asthma; and even areca was occasionally of use in cases of tapeworm, where male-fern had failed.

Of the 114 new remedies or preparations added, upon most it is simply setting the seal of official sanction, as they have been already brought into familiar use in daily practice. Lamellæ, or "discs," are now officinal, prepared with cocaine, physostigmine, and atropine; and "tabellæ nitro-glycerini" are also introduced: these are defined as tablets of chocolate, containing $\frac{1}{100}$ grain of pure nitro-glycerine. These tablets, we must suppose, fall from heaven (like manna), all ready for use; for the editors do not condescend to tell us what either nitro-glycerine or chocolate is, or how they can be recognised, or how these tablets are to be made, or what is to be done with them when they are thus miraculously obtained!

Upon a few of the individual additions we must make a brief remark. Apomorphia is introduced in the form of the hydrochlorate; and a solution for hypodermic injection is given, which "must be made as required for use:" this is very inconvenient, and would be obviated by having lamella of this alkaloid also. The use of apomorphia, except by the hypodermic method, is not alluded to, nor any dose stated, although, given by the mouth, it is a valuable expectorant in cases where the sputum is viscid. Cocaine is presented to us in the form of lamella only, although it is desirable to have an officinal solution of, say, 4 per cent. strength. Under the elegant Latinity of ext. cascar*æ* sagrad*æ*, a preparation of the rhamnus purshianus is now recognised, as well as similar preparations of the rhamnus frangula. As these remedies are now in constant use, we think this is a step in the right direction.

The introduction of the active principle elaterin, in place of the uncertain substance elaterium, in the composition of the compound powder of the drug, is also a right step; but the doses given of elaterin and of pulv. elaterini co. do not tally with one another. Jaborandi, and its alkaloid, pilocarpine, in the form of the nitrate, are placed in the new Pharmacopœia. The galenical preparations of jaborandi seem uncertain in their action; perhaps on account of the presence of the countervailing alkaloid, jaborine, which exists also in varying quantities in the leaves of the plant. Pilocarpine is, therefore, much to be preferred, and is usually administered by hypodermic injection. The editors do not specify whether the doses which they recommend are intended to be given by the mouth or otherwise: the maximum dose mentioned ($\frac{1}{2}$ grain) is certainly too large to be safely administered by the skin. Seeing how frequently ext. belladonnæ is prescribed for internal use, the introduction of another extract without a very distinctive name is likely to prove another source of dangerous mistakes. The large development of antiseptics since the publication of the previous edition of the *British Pharmacopœia* is fairly recognised by the introduction of several fresh antiseptic substances. Boric, salicylic, and chromic acids, iodoform, menthol, thymol, eucalyptus oil, and the sulpho-carbolates of zinc and sodium, are now added, and most of them rightly so. Two new remedies are introduced, of which nothing but disapprobation can be expressed: actæa racemosa, now called cimicifuga, which experience long ago showed to be completely useless; and staphisagria, which, though certainly a potent destroyer of pediculi, is not more so than other well-known substances, and is a violent and dangerous poison. In the preface to the present edition it is stated that "pains have been taken to bring the whole of the matter up to the existing state of knowledge;" but if that is so, why are we not provided with certain remedies of more value, and in more general use, than cimicifuga, for example? Why are not euonymin, convallarin, hamamelis, and homatropin, *inter alia*, placed in the new Pharmacopœia? Of the newer anti-pyretics, kairin, anti-pyrin, thallin, and such like, we must presume the editorial committee have not yet heard, though they are, and will continue to be, largely used, M. Jacoud's statements notwithstanding. In conclusion, it is to be regretted that, having plagiarised the Pharmacopœia of the United States so largely, the General Medical Council should not have had the courtesy to acknowledge the source from which nearly all their valuable innovations have been derived.

